# MicroRNA-145 suppresses epithelial to mesenchymal transition in pancreatic cancer cells by inhibiting TGF-β signaling pathway

**DOI:** 10.7150/jca.34902

**Published:** 2020-02-19

**Authors:** Shaojun Chen, Junyi Xu, Yao Su, Li Hua, Chengjun Feng, Zhan Lin, Haixin Huang, Yongqiang Li

**Affiliations:** 1 Department of Oncology, the Forth Affiliated Hospital of Guangxi Medical University, Liuzhou, Guangxi, China; 2 Department of general surgery, The Fourth Affiliated Hospital of Guangxi Medical University, Liuzhou, Guangxi, China.; 3 Nanjing Zhongshan Biomedical Translational Institute, Nanjing, Jiangsu, China; 4 Department of Oncology, The Yulin First People's Hospital , Yulin, Guangxi ,China; 5 Department of Chemotherapy, the Affiliated Tumor Hospital of Guangxi Medical University, Nanning, Guangxi, China

**Keywords:** miRNA-145, pancreatic cancer, EMT, metastasis, TGF- β signaling

## Abstract

TGF-β signaling plays a critical role in tumor progression and many approaches have been made to inhibit its functions. MicroRNA is one of the approaches that inhibit TGF-β signaling and can be used as a promising treatment for cancer. This study explored the role of miRNA-145 in pancreatic cancer (PC) development. The expression of miRNA-145 in PC tissues and paired adjacent normal tissues was examined by qRT-PCR. The expression of miRNA-145 in PC cells and the ability of cell migration and invasion were detected both *in vivo* and *in vitro*. The results showed that miRNA-145 was down-regulated in PC tissues and PC cells. Increasing the expression of miRNA-145 in PC cells inhibited the TGF-β signaling pathway and epithelial-mesenchymal transition (EMT) process. Scratch assay and transwell assay showed that miRNA-145 inhibited the migration and invasion in PC cells. *In vivo* experiments confirmed that miRNA-145 mimics delayed the growth of PC xenografts comparing with miRNA-145 inhibitor. Our results suggested that miRNA-145 can inhibit epithelial to mesenchymal transition (EMT) and tumor growth by suppressing TGF-β signaling pathway. Thus, miRNA-145 could be a potential therapeutic for targeting TGF-β signaling in PC treatment.

## Introduction

Pancreatic cancer (PC) is a highly aggressive malignant tumor which is the fourth most common cause of cancer related death in Western countries^1^. The five-year survival rate of PC is less than 5%^2^. PC is difficult to be diagnosed at early stages, because the early clinical symptoms of PC are not typical. Currently, there was no effective drug and other treatment for PC. Thus, exploring molecular mechanisms of PC tumorigenesis would provide valuable insights to improve its prognosis.

MicroRNAs (miRNAs) are a class of highly conserved, endogenous non-coding small RNAs containing approximately 21 to 23 nucleotides^3^, ^4^. By complementary binding to the 3'-untranlated region (3' UTR) of target mRNA, miRNAs could degrade the target mRNAs or inhibit the translation of the target mRNAs, leading to regulate cell proliferation, differentiation, invasion, migration and cell death^5^, ^6^. miRNA-145 was reported as a tumor suppressor, because it showed low expression in different types of tumors and its expression was correlated with the progression of tumors, including breast cancer, lung cancer and colorectal cancer^7^-^9^. Studies showed that miR-145 was capable of binding to the 3'-UTR of SMAD3, thereby inhibiting its protein expression and subsequent inhibiting the transforming growth factor beta (TGF-β)-induced epithelial to mesenchymal transition (EMT) 10, 11. However, miR-145 could promote the EMT in human peritoneal mesothelial cells by restraining the fibroblast growth factor 10 12. The mechanism of miR-145 needs to be elucidated in the EMT process.

TGF-β is a member of the transforming growth factor beta superfamily of cytokines. Many studies have established that TGF-β induces EMT in cancer cells^10^. When binding to its membrane receptor, TGF-β activated its downstream factors, including Smad2, Smad3 and Smad4, leading to EMT 11. For instance, TGF-β triggered EMT by reducing epithelial biomarkers such as E-cadherin and accumulating of mesenchymal biomarkers such as vimentin 15, 16. Some studies had demonstrated that miR-145 can suppress TGF-β induced EMT and attenuate cell migration and invasion 12. However, the association between miRNA-145 and TGF-β-induced EMT remains unclear in pancreatic cancer.

In this study, we up- and down-regulated the expression of miRNA-145 in PC cells and evaluated the expression of EMT related biomarkers in these cells. The ability of cell invasion and migration were examined by scratch assay and transwell assay. A pancreatic tumor xenograft mouse model was used to confirm the effects of miRNA-145 in tumor growth *in vivo*.

## Methods

### Cell lines

The human pancreatic duct epithelial cell line HPDE6c7 was purchased from Kerafast (Boston, USA) and cultured in keratinocyte serum-free medium with human recombinant epidermal growth factor (EGF1-53) and bovine pituitary extract (BPE). Three pancreatic cancer cells PANC-1, SW1990 and BxPC-3 were purchased from cell bank of the Chinese Academy of Sciences (Shanghai, China), and cultured in DMEM containing 10% FBS in a humidified atmosphere with 5% CO_2_. All the cells used in this study were in the exponential growth phase.

### Tissue samples collection

A total of 42 cases of paired primary PC tissues and their adjacent normal tissues (from the border of cancer tissue ≤3cm) were collected from PC patients who had been histopathologically and clinically diagnosed in the Forth Affiliated Hospital of Guangxi Medical University between 2014 and 2017. All the patients were not treated with any anti-cancer therapy before surgical resection. Tissue samples were quickly frozen in liquid nitrogen after surgery and stored in -80°C freezer. This study was approved by the institutional ethics committee of the Forth Affiliated Hospital of Guangxi Medical University and all patients signed written informed consents.

### RNA extraction, cDNA synthesis, and real-time quantitative reverse transcriptase PCR (qRT-PCR)

Total RNA was isolated from tissue samples or cell pellets using Trizol reagent (Invitrogen, Carlsbad, USA). After reverse transcription, cDNA was synthesized and used as the template for qPCR. The volume of each reaction was 10μL: SYBR green Master 5μL, 10μM forward primer 0.5μL, 10μM reverse primer 0.5μL, cDNA 1μL, nuclease-free water 3μL. The cycling mode was as follows: initial denature at 95°C for 30sec, denature at 95°C for 5s and anneal at 60°C for 30s. The PCR reaction was carried out for a total of 40 cycles.

### Western blotting

Total proteins were isolated from cells by using RIPA lysis buffer containing protease cocktail and PMSF. After quantification, 25μg protein was loaded into the 10% SDS-PAGE gel and separated under electrophoresis. Then the separated proteins were transferred onto PVDF membrane. After blocking by 5% non-fat milk, the membrane was incubated with different primary antibodies including Vimentin (#49636, 1:1000, Cell Signaling Technology), E-cadherin (#14472, 1:1000, Cell Signaling Technology), TGF-β ( #3709, 1:1000, Cell Signaling Technology), p-SMAD2 (#3104, 1:1000, Cell Signaling Technology) and SMAD2 (#5339, 1:1000, Cell Signaling Technology), followed by the HRP-conjugated secondary antibodies (1:5000, Cell Signaling Technology). ChemiDoc imaging machine (Bio-Rad, Berkeley, USA) was used to visualize target proteins with ECL assay.

### miRNA-145 transfection

MiRNA-145 mimics and inhibitor were purchased from Sangon Biotech (Shanghai, China). PC cells were transfected with100mM mimics, inhibitor or its corresponding scramble control miR-NC using Lipofectamine 2000 (Invitrogen, Carlsbad, California, USA) according to manufacturers' instruction. After 48h, the cells were used for experiments. Each experiment was performed in biological triplicate.

### MTT assay

About 3×10^4^ cells were seeded into 96-well plate. After overnight culturing, 20μL MTT (5mg/ml) was added to each well and continued to culture for 4h. Then 150μL DMSO was added to each well and the plate was read by a spectrometer (Bio-rad, Berkeley, USA).

### Scratch assay

Cells were seeded into 6-well plate with the concentration of 3×10^5^/ml. After overnight culturing, wounds were created by using a 200μL pipet tip. Then the medium was changed to remove the detached cells. After culturing for 48h, image was captured by an inverted microscopy (Nikon, Tokyo, Japan) and the wound healing ability of each cell line was analyzed.

### Transwell assay

The transwell chambers (Corning, NY, USA) with Matrigel were used to detect cell invasion and chambers without Matrigel were used to examine cell migration. 5 × 10^4^ cells were seeded into the upper chambers of transwell in 200 µL serum-free medium. 500 µL medium containing 10% FCS was used as the chemo-attractant and added to the lower wells. After 24h, the chambers were fixed with 80% ethanol and stained with crystal violet (20mg/ml). Then the cell numbers were counted in five individual random fields (200×) under a light microscope (Olympus, Tokyo, Japan), and the average cell density per field was calculated.

### Pancreatic cancer mouse xenograft model

All the procedures of animal experiments were approved by the Animal Care and Use Committee of the Forth Affiliated Hospital of Guangxi Medical University. 20 immunodeficient mice (BALB/c) were randomly separated into four groups: control group, miR-145 inhibitor group, miR-145 mimic group and SMAD7 group. After anesthesia, 5×10^6^ cells were subcutaneously implanted into each immunodeficient mouse. Tumor samples were collected at 21d after implantation.

### Statistical analysis

All data was analyzed with the statistical software SPSS (version 21). All quantitative data was displayed as mean ± SD and analyzed using Student t-test, while ratio data was analyzed with the X^2^ test. A value of P<0.05 was considered as statistically significant.

## Results

### Low expression of miRNA-145 in pancreatic cancer tissues and cells

**Figure 1:**
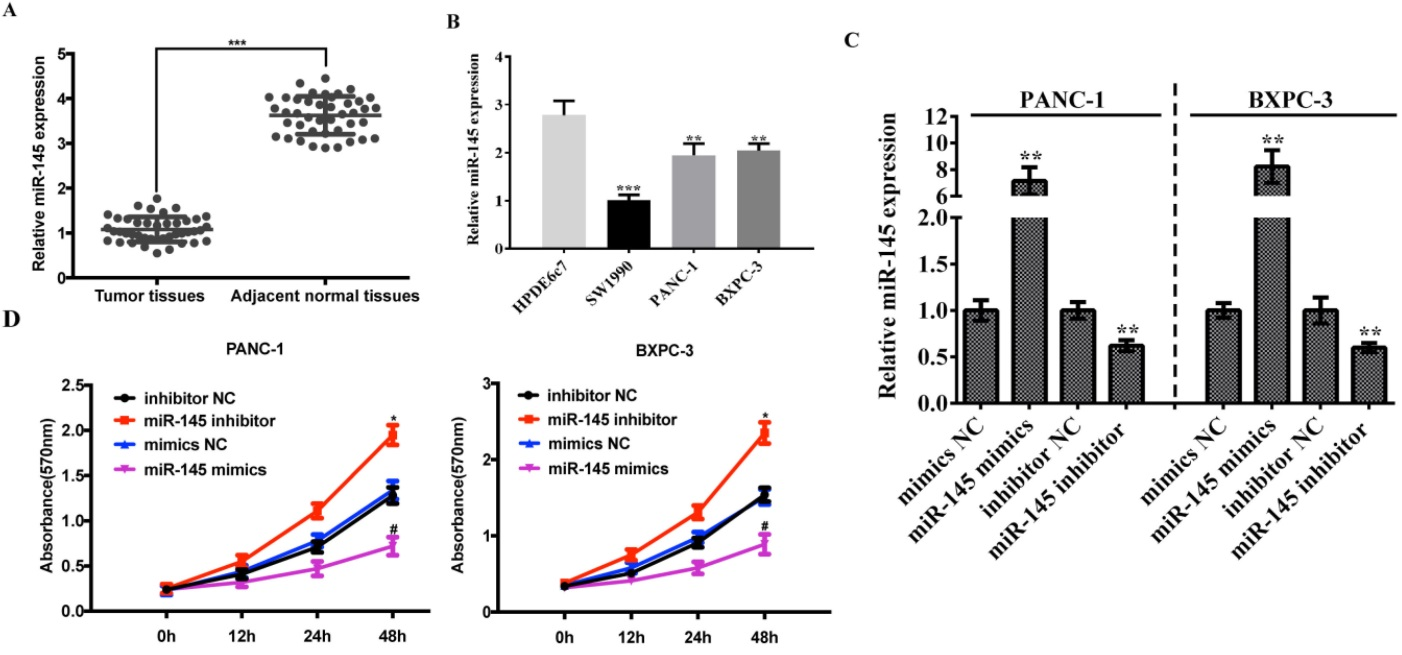
** MicroRNA-145 is down-regulated in PC tissues and cells.** qRT-PCR on the expression of miRNA-145 in ( A): PC tissues and the paired adjacent normal tissues; (B): PC cells; and (C): PANC-1 and BxPC-3 cells transfected with mimic and inhibitor; D: MTT assay on the proliferation of PC cells after transfected with miRNA-145 mimic and inhibitor.

In order to clarify the association between miRNA-145 and PC, we examined the expression of miRNA-145 in 42 cases of paired primary PC tissues and their adjacent normal tissues using qRT-PCR. The results showed a significantly lower level of miRNA-145 expression in PC tissues compared with the paired adjacent normal tissues (Fig 1A). Meanwhile, the expression of miRNA-145 was also examined in different PC cell lines, and the qRT-PCR results showed a low expression of miRNA-145 in all three human pancreatic cancer cell lines—PANC-1, SW1990 and BxPC-3 when compared with the human pancreatic duct epithelial cell line HPDE6c7 (Fig 1B). Two cell lines, PANC-1 and BxPC-3 that had a relatively high expression miRNA-145, were chosen for further research.

In order to further identify the function of miRNA-145 in PC cells, we modified the expression level of miRNA-145 in these two cell lines. PANC-1 and BxPC-3 cells were transfected with miRNA-145 mimics or inhibitor and the efficiency of transfection was verified by qRT-PCR. The results showed that the mimics up-regulated the miRNA-145 expression while the inhibitor down-regulated the expression in both cell lines (Fig 1C). MTT was performed to detect the cell proliferation and the results showed that PC cells transfected with miRNA-145 inhibitor proliferated faster than the cells transfected with the scramble control, while miRNA-145 mimics delayed the cell proliferation comparing with the scramble control (Fig 1D).

### MicroRNA-145 inhibited TGF-β signaling pathway in PC cells

**Figure 2:**
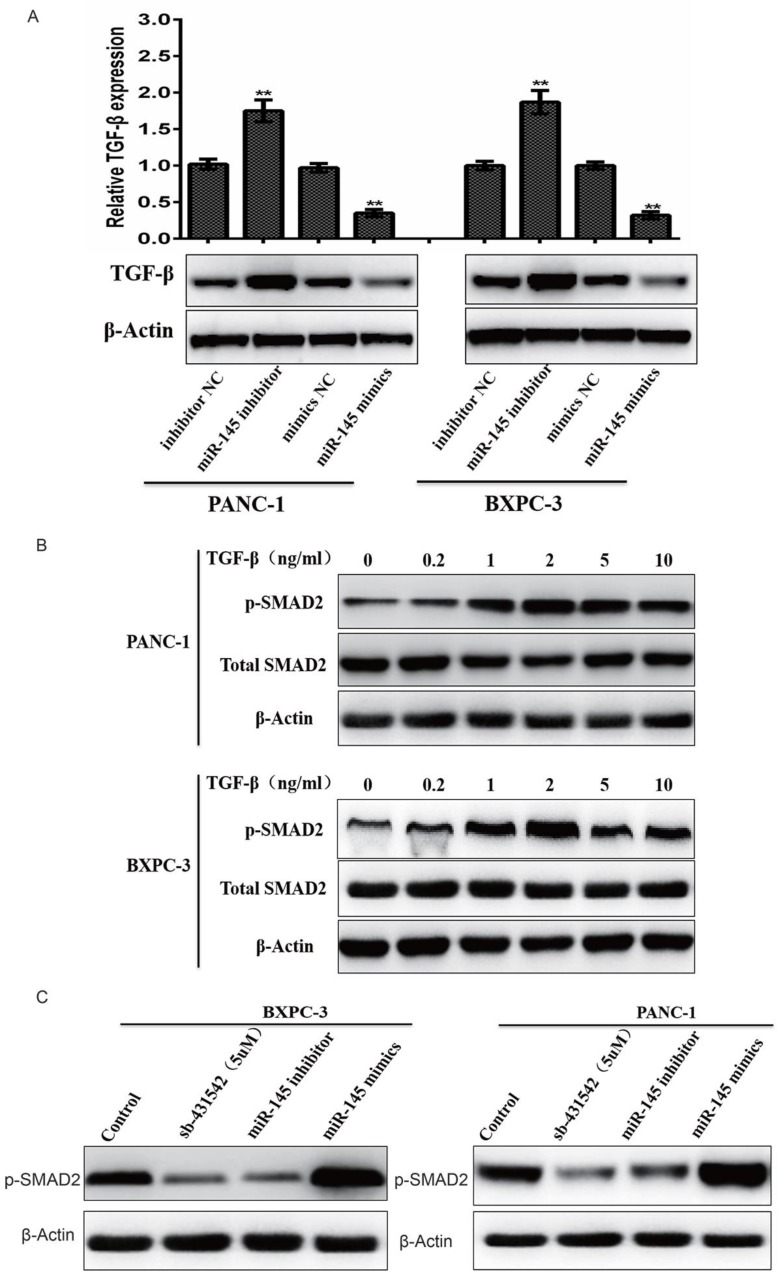
** MicroRNA-145 inhibits TGF-β signaling pathway in PC cells.** Western blotting. A: TGF- β expression after transfected with miRNA-145 mimic and inhibitor in PC cells; B: the expression of p-smad-2 in PC cells treated with different concentration of TGF- β; C: the expression of p-smad2 in PC cells transfected with miRNA-145 mimic and inhibitor.

In order to understand the relationship between miRNA-145 and TGF- β signaling pathway, biomarkers related to TGF- β signaling were evaluated in PC cells after transfected with miRNA-145 mimic or inhibitor. Western blotting results showed that TGF- β expression was up-regulated in PANC-1 and BxPC-3 cells transfect with miRNA-145 inhibitor when compared to the cells transfected with scramble control. In contrast, miRNA-145 mimics down-regulated TGF- β expression in these two types of cells (Fig 2A). Treatment of cells with different concentration of TGF- β activated TGF- β signaling in PC cells, that manifested as the high expression of p-Smad2 protein and this effect was dose-dependent (Fig 2B). The expression of p-Smad2 was examined in PANC-1 and BxPC-3 cells with altered expression level of miRNA-145. It was found that p-Smad2 expression was down-regulated in these two cell lines when transfected with miRNA-145 mimics, and the effect is comparable to that of treatment by 5μM sb-431542, a well- known TGF- β inhibitor. On the other hand, p-Smad2 expression was up-regulated in cells transfected with miRNA-145 inhibitor (Fig 2C). These results indicated that miRNA-145 inhibited TGF- β signaling pathway.

### MicroRNA-145 inhibited EMT in PC cells

**Figure 3:**
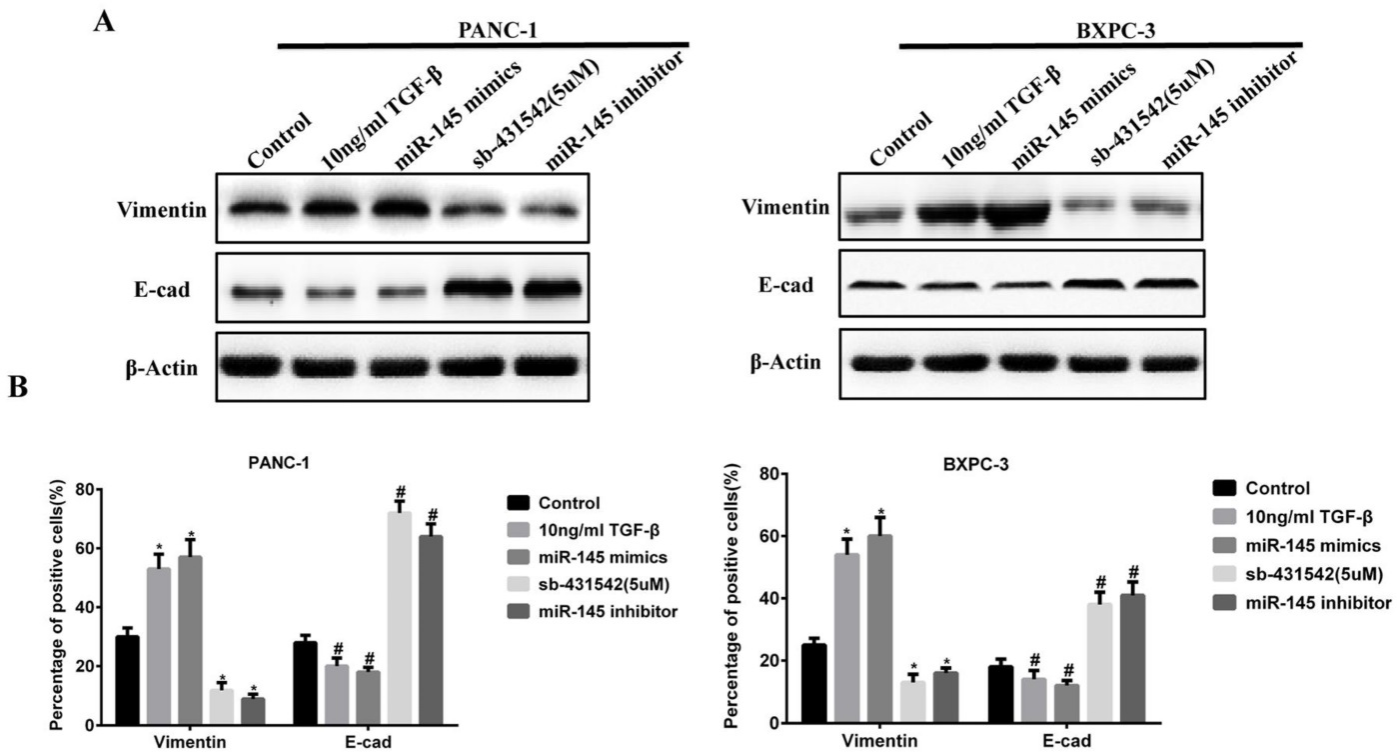
** MicroRNA-145 inhibits EMT in PC cells.** A: Western blotting and B: qRT-PCR on the expression of epithelial and mesenchymal biomarkers in PC cells transfected with miRNA-145 mimic and inhibitor.

The above results indicated that miRNA-145 could inhibit TGF-β signaling pathway in PC cells. Given that TGF-β signaling pathway promotes EMT process, we examined the change of expression of EMT related biomarkers. During EMT, the expression of epithelial cell biomarkers decreased while that of mesenchymal cells increased. We examined the expression of epithelial biomarker E-cadherin and mesenchymal biomarker vimentin in PC cells after transfection with miRNA-145 mimic or inhibitor. Western blot and qRT-PCR results showed that the expression of vimentin increased while that of E-cadherin decreased in PANC-1 and BxPC-3 cells after transfection with miRNA-145 inhibitor, and the effects were similar to 10ng/ml TGF-β treatment. In contrast, the opposite effects were observed in PC cells transfected with miRNA-145 mimics (Fig 3A and B).

**Figure 4:**
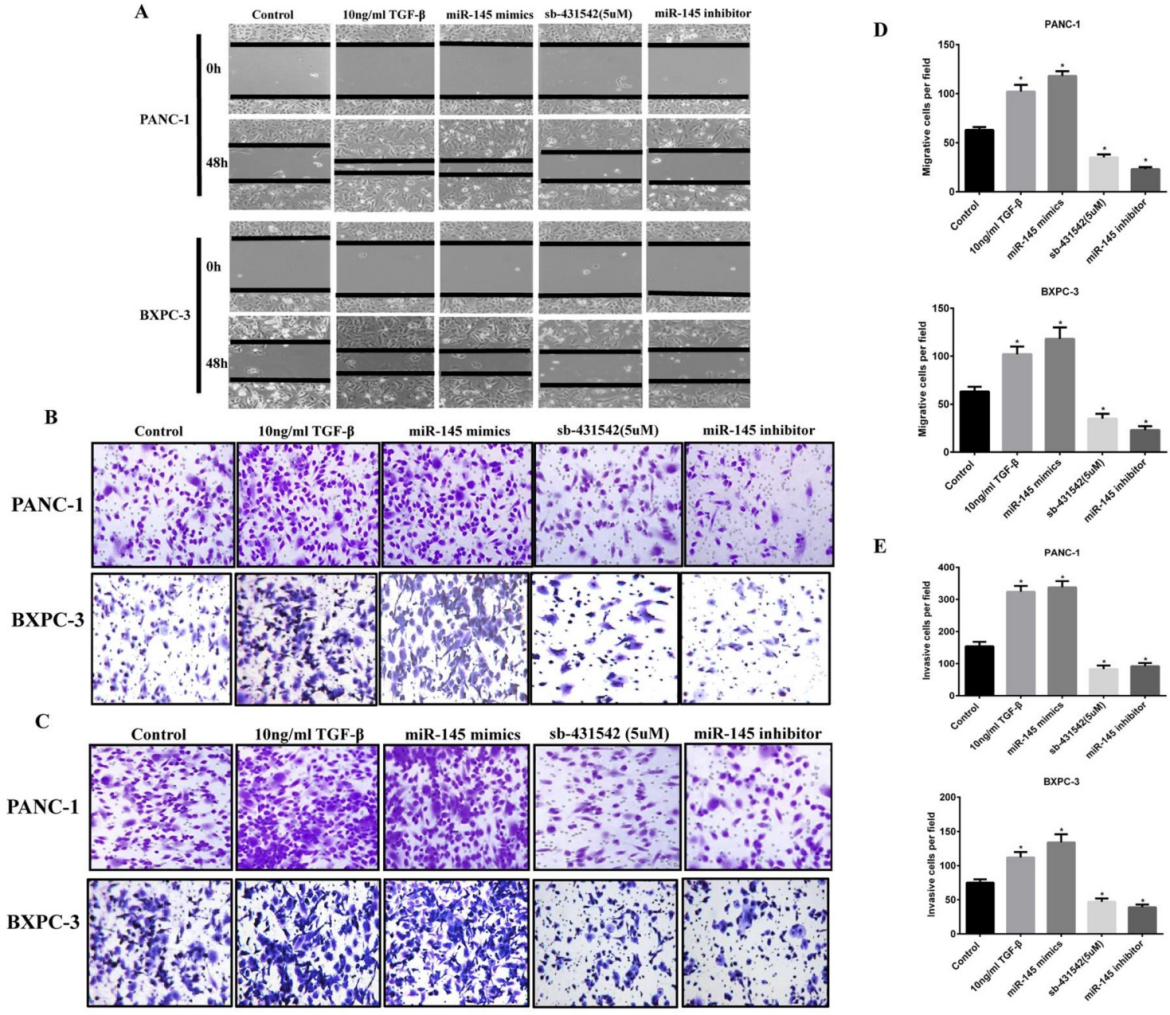
** MicroRNA-145 inhibits cell migration and invasion in PC cells.** Cell migration and invasion of PC cells transfected with miRNA-145 mimic and inhibitor examined by A: Scratch assay; B: Transwell assay; and C: Transwell assay with matrigel. D: The quantitative analysis of transwell assay; E: The quantitative analysis of transwell assay with Matrigel.

Wound healing experiments showed that the scratch healing ability of PC cells transfected with miRNA-145 inhibitor was significantly higher than that of cells transfected with the scramble control after 48h, the wounded area was much smaller than that in the miR-145 inhibitor group(Fig 4A). Meanwhile, cell migration and invasion were detected by Transwell assay. The results showed that the number of cells in the group of miRNA-145 inhibitor migrated and invaded to the bottom of the chamber was significantly increased. And the effect of miRNA-145 inhibitor was similar to that of 10ng/ml TGF-β treatment. The opposite results were found in the PC cells transfected with miRNA-145 mimic which demonstrated low ability of wound healing, as well as cell migration and invasion (Fig 4B and C).

### MicroRNA-145 inhibited pancreatic cancer growth *in vivo*

**Figure 5:**
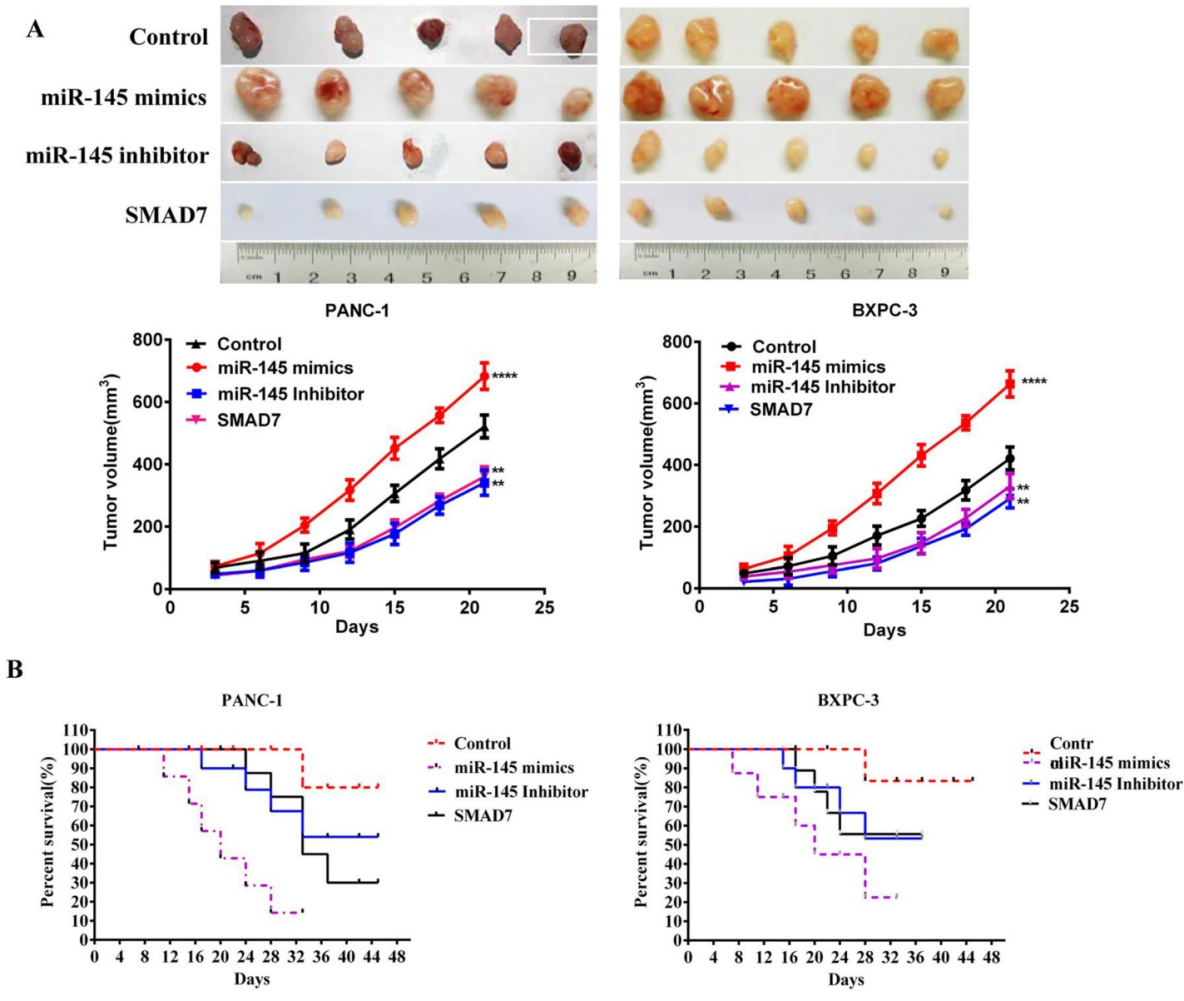
** MicroRNA-145 inhibits pancreatic cancer metastasis *in vivo.***A: Tumor size of xenografts with PC cells transfected with miRNA-145 mimic and inhibitor; B: The Kaplan-Meier curve of xenograft with PC cells transfected with miRNA-145 mimic and inhibitor.

PANC-1 cells were transfected with miRNA-145 mimics, inhibitor, scramble controls or SMAD7 (a negative regulator of TGF- β signaling) expression plasmid in order to obtain the cells with different proliferation potentials. Then the cells were subcutaneously injected into immunodeficient mice to establish xenografts of prancreatic cancer. The tumor size was measured at 21d after cell inoculation. The results showed that PC cells transfected with miRNA-145 inhibitor leading to significant increases in tumor volume and weight (*P*<0.05), while cells with miRNA-145 mimics significantly inhibited the tumor growth (Fig 5A).

The Kaplan-Meier curve showed that the survival of mice transplanted with miRNA-145 mimics PANC-1 cells was longer than that of mice transplanted with miRNA-145 inhibitor PANC-1 cells (Fig 5B), demonstrating that miRNA-145 inhibited PC proliferation *in vivo.*

## Discussion

The dysregulation of miRNA-145 expression has been reported in different types of cancers. In bladder cancer and lung cancer, miRNA-145 inhibits migration and invasion of tumor cells by directly targeting N-cadherin^12^, ^13^. In the present study, we found that the expression of miRNA-145 was down-regulated in PC tissues comparing to the paired adjacent no cancerous tissues. Furthermore, PC cell lines also showed low expression of miRNA-145, indicating the correlation between miRNA-145 expression and pancreatic cancer.

TGF-β is a multifunctional cytokine belonging to the transforming growth factor superfamily. When binding to its receptor, TGF-β activates a signaling cascade and leads to multiple outcomes, including cell differentiation, cell proliferation and chemotaxis^14^, ^15^. TGF-β has different effects in cancers, it acts as a tumor suppressor in premalignant cancers and a tumor promoter in advanced cancers, especially in the invasion and metastasis 16, 17. TGF- β signaling has been confirmed to accelerate PC progression^18^ and the relationship between microRNA and TGF- β signaling has been extensively studied^19^, ^20^. In this study, we up-regulated miRNA-145 expression in PANC-1 and BxPC-3 cells, that resulted in the down regulation of TGF-β signaling. According to the TargetScan (http://www.targetscan.org/), TGF-β receptors and smad2 were the direct targets of miRNA-145. Thus, the high expression of miRNA-145 led to the degradation of TGF-β receptors and SMAD2, following by the deactivation of TGF-β signaling.

Previous studies confirmed the relationship between TGF-β signaling and EMT. TGF-β induced EMT in different epithelial cells* in vitro* 21 by activating Snail, ZEB and bHLH families, that playing critical roles in the EMT process^22^. This phenomenon also existed in PC cells^23^. It was shown that miR-145 could bind to the 3'-UTR of SMAD3 and metadherin mRNA to inhibit protein expression, thereby repressing the TGF-β-mediated EMT process including cancer cell metastasis and invasion 10, 11. In our study, TGF-β stimulated EMT in two PC cell lines. When the expression of miRNA-145 in PC cells was down-regulated, it led to the up-regulation of TGF-β signaling and promoted the EMT process. In addition, increased abilities of cell migration and invasion were observed when the expression of miRNA-145 in PC cells was down regulated. The opposite effects were found in PC cells transfected with miRNA-145 mimics since TGF-β signaling was inhibited. These results indicated that miRNA-145 inhibits EMT in PC cells through TGF-β signaling. *In vivo* experiments also supported that miRNA-145 could suppress the tumor growth of PC cells.

In summary, our results suggested that the expression of miRNA-145 was down-regulated in PC tissues comparing to paired adjacent normal tissues. Down-regulation of miRNA-145 activated TGF-β signaling, followed by enhanced abilities of cell migration and invasion both *in vivo* and *in vitro.* The role of miRNA-145 in inhibiting EMT through TGF-β signaling pathway indicated that miRNA-145 could be a potential candidate in anti-cancer drug development.
